# The Role of Glenoid Osteotomy in the Treatment of Shoulder Dysplasia in Brachial Plexus Birth Palsy: A Systematic Review of the Literature

**DOI:** 10.3390/jcm14165610

**Published:** 2025-08-08

**Authors:** Chiara Arrigoni, Roberto Facchi, Nunzio Catena

**Affiliations:** 1 Hand Surgery and Reconstructive Microsurgery, IRCCS Istituto Giannina Gaslini, Via Gerolamo GAslini 5, 16147 Genova, Italy; chiaraarrigoni@gaslini.org; 2 Department of Orthopedics and Traumatology, IRCCS San Martino, University of Genoa, 16147 Genova, Italy; roberto.facchi94@gmail.com; 3 Hand Surgery and Reconstructive Microsurgery Unit, IRCCS Istituto Giannina Gaslini, Via Gerolamo Gaslini 5, 16147 Genova, Italy

**Keywords:** glenoid osteotomy, brachial plexus birth injury, shoulder dysplasia, pediatric shoulder surgery, functional outcomes

## Abstract

The treatment of shoulder dysplasia resulting from brachial plexus birth injury (BPBI) remains a matter of debate within pediatric orthopedic and neurosurgical communities. Various approaches have been proposed to address the muscular imbalance and joint incongruity that develop in affected children, with special attention paid to the roles of humeral head reduction and tendon transfers. **Background/Objectives**: These procedures aim to correct the disproportionate strength between internal and external rotators of the shoulder. However, the specific contribution of skeletal procedures such as glenoid osteotomy to restoring shoulder mechanics remains controversial. Glenoid osteotomy, a technique that involves surgically reorienting the glenoid cavity, is hypothesized to promote better containment of the humeral head and allow more physiological joint development. On one hand, altering the glenoid axis could enhance joint congruency and facilitate remodeling during growth. On the other hand, there is limited evidence supporting its efficacy and safety. **Methods**: This review aims to assess the available literature to determine whether glenoid osteotomy represents a safe and effective procedure for patients with BPBI-associated shoulder dysplasia. A comprehensive literature search yielded 1380 titles. After excluding studies focused on adults and those failing to meet inclusion criteria, only three studies were selected for final analysis. Due to the limited data and variability in study design, no statistical meta-analysis could be performed. **Results**: Findings suggest that glenoid osteotomy, particularly when combined with tendon transfers, may lead to improvements in shoulder abduction and external rotation. However, outcomes are often difficult to interpret in isolation, and the specific benefits attributable to the osteotomy remain unclear. The lack of standardized imaging, follow-up, and scoring systems limits the strength of current conclusions. **Conclusions**: Further multicenter, prospective studies are needed to evaluate the long-term efficacy of glenoid osteotomy, its role in skeletal remodeling, and its contribution to overall shoulder stability and function. Such studies would help clarify the true potential of this surgical technique in the broader context of BPBI treatment.

## 1. Introduction

Brachial plexus birth injury (BPBI) is a perinatal nerve trauma that involves injury to the cervical nerve roots comprising the brachial plexus. It typically occurs during difficult or traumatic deliveries and is most frequently associated with obstetric complications such as shoulder dystocia, fetal macrosomia, prolonged second-stage labor, or breech presentation.

Despite the evolution of obstetric techniques and the implementation of preventive strategies, BPBI continues to represent a significant cause of neonatal neuromuscular disability worldwide.

Clinically, BPBI presents with a heterogeneous spectrum of neurological deficits. The severity and distribution of the injury are commonly classified according to the Narakas system, a widely accepted framework based on the extent of nerve root involvement. In Narakas type I, only the upper roots (C5–C6) are affected; in type II, the injury extends to the C7 root, while type III and IV involve the whole plexus (type IV adds signs of Horner’s syndrome—such as ptosis and miosis—due to damage to the sympathetic chain).

In the early stages, many infants—particularly those with types I and II lesions—exhibit spontaneous nerve regeneration, often within the first three to six months of life.

Nerve repair with grafts or nerve transfer ensures muscle recovery in a large group of patients who did not recover spontaneously, especially those of type II, III, and IV [1].

However, a substantial subset of patients continues to experience persistent deficits, particularly in shoulder function. The glenohumeral joint, due to its high degree of mobility and its reliance on balanced muscular control, is especially vulnerable to long-term sequelae. In cases where there is an imbalance between overactive internal rotators—such as the subscapularis and pectoralis major—and weakened or paralyzed external rotators like the infraspinatus and teres minor, the shoulder joint becomes predisposed to progressive structural deterioration.

Over time, these biomechanical alterations give rise to shoulder dysplasia (SD), a pathological state characterized by joint malformation and instability.

The defining anatomical features of SD include glenoid retroversion, flattening or erosion of the articular surface, posterior migration of the humeral head, and—particularly in advanced cases—the development of a biconcave glenoid configuration. These deformities severely restrict both passive and active motion, especially in external rotation and abduction, and contribute to compromised upper limb function during growth and maturation [2].

To objectively assess the degree of glenohumeral deformity, clinicians frequently use the Waters classification system, which grades the severity of joint pathology from type I (normal or near-normal anatomy) to type VII, which indicates complete posterior dislocation of the humeral head from the glenoid. This radiographic system assists in standardizing clinical assessment and plays a crucial role in informing therapeutic strategies, particularly when considering surgical options [3].

In mild dysplasia cases (Waters I–II), where the joint remains relatively congruent and deformity is minimal, soft tissue procedures often suffice. These procedures aim to optimize shoulder kinematics without altering the bony structure.

On the other hand, in more severe dysplasia (Waters V–VII), where structural alterations are profound and joint congruency is significantly compromised, bony interventions become necessary. Among these, humeral derotational osteotomy is commonly performed to realign the humerus and compensate for internal rotation contractures. Yet such procedures are largely salvage-oriented, addressing symptoms rather than reversing the underlying pathology [4,5,6,7,8].

The intermediate stages of SD (Waters III–IV) present a unique therapeutic dilemma. In these cases, the joint is malformed but still retains potential for remodeling. It is in this clinical context that glenoid osteotomy has gained attention as a possible surgical solution. The goal of this procedure is to reorient the glenoid cavity into a more anatomically favorable, anteverted position. The hypothesis is that such correction can improve humeral head containment, restore joint congruity, and thereby enhance the long-term success of concurrent soft tissue procedures like tendon transfers or open joint reductions.

Despite its conceptual appeal, the clinical application of glenoid osteotomy remains limited and is associated with several challenges.

This review was therefore undertaken to synthesize and critically appraise the current literature on glenoid osteotomy in the setting of BPBI-associated shoulder dysplasia. By systematically examining indications, surgical techniques, functional outcomes, and reported complications, we aim to clarify whether glenoid osteotomy represents a viable and beneficial component of the contemporary surgical algorithm for managing this complex pediatric condition.

## 2. Materials and Methods

To assess the existing evidence regarding the efficacy and safety of glenoid osteotomy in pediatric patients with shoulder dysplasia secondary to brachial plexus birth injury (BPBI), the authors conducted a review of the literature. This research aimed to identify relevant studies that reported clinical outcomes following glenoid reorientation procedures, either as standalone interventions or in combination with other surgical techniques.

A comprehensive search strategy was developed and executed across five major electronic medical databases: PubMed, Embase, Web of Science, Scopus, and the Cochrane Library.

This review was registered on PROSPERO International Prospective Register of Systematic Review (PROSPERO 2025 CRD420251115161).

The searches were not limited by publication year or article type in order to capture the widest possible range of relevant publications until December 2024. Only articles published in English were considered.

The authors adopted this strategy because, as glenoid osteotomy is not widely used, it was felt that there were no relevant publications outside the English-language literature.

The primary search term used was “glenoid osteotomy” in order to obtain the widest possible list of publications.

All the databases listed above were consulted according to the following scheme:
**Database****Search Terms****Filters****Results**Embase(‘glenoid’/exp OR glenoid) AND (‘osteotomy’/exp OR osteotomy) AND [english]/lim AND [embase]/limEnglish398-20 December 2024PubMed(“glenoid” [All Fields] OR “glenoidal” [All Fields] OR “glenoids” [All Fields]) AND (“osteotomie” [All Fields] OR “osteotomied” [All Fields] OR “osteotomy” [MeSH Terms] OR “osteotomy” [All Fields] OR “osteotomies” [All Fields])
270-20 December 2024Scopus(TITLE-ABS-KEY (osteotomy)) AND (TITLE-ABS-KEY (glenoid))
439-20 December 2024Web of ScienceTopic Search = osteotomy AND Topic Search = glenoidEnglish265-20 December 2024CochraneALL = glenoid ALL = osteotomy #1 AND #2English7 trials + 4 systematic reviews

The inclusion criteria were clearly defined prior to initiating the search. Studies were eligible for inclusion if they met all of the following conditions:

- Included pediatric patients (under the age of 18) with a diagnosis of BPBI;
- Described the use of glenoid osteotomy as part of the surgical management;
- Reported clinical or radiological outcomes.

Described whether the osteotomy was performed in isolation or combined with other procedures such as tendon transfers, soft tissue releases, or open joint reductions. Exclusion criteria included:

- Studies focusing solely on adult patients;
- Reports lacking outcome data or clear methodology;
- Case reports with fewer than three patients;
- Review articles, editorials, and letters to the editor.

From an initial yield of 1380 titles, two independent reviewers (CA and RF) conducted an initial screening of titles and abstracts. Of these, 1342 articles were excluded because they primarily addressed shoulder pathology in adults or did not involve surgical intervention. The remaining 38 abstracts were assessed in detail, leading to the exclusion of an additional 31 studies based on the previously stated criteria.

**Figure 1:**
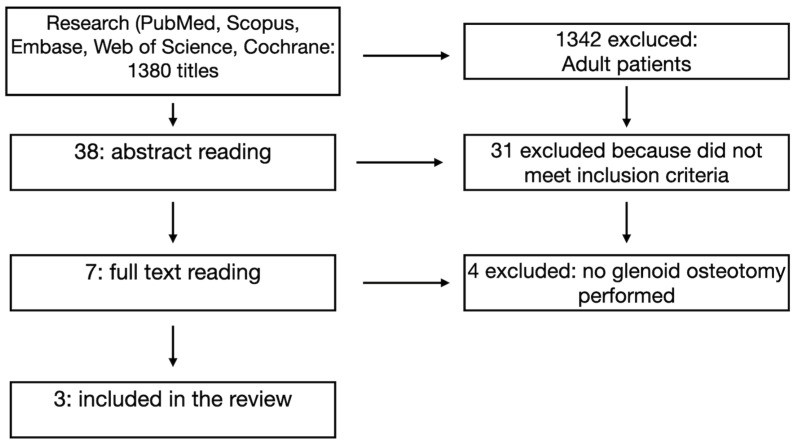
PRISMA flow diagram of inclusion criteria and qualitative analysis.

The full texts of the final 7 articles were retrieved and thoroughly analyzed by two of the authors (CA and NC). Ultimately, only 3 studies met all inclusion criteria and were retained for qualitative analysis (as summarized in Figure 1).

The quality of the included papers was assessed with ROBINS—I Tools, finding a moderate risk of bias.

The limited number of studies and significant heterogeneity in study design, patient population, and outcome measures precluded the possibility of performing a quantitative meta-analysis or formal statistical comparisons.

Data were extracted from the included studies regarding patient demographics, surgical techniques, perioperative imaging, follow-up duration, and outcome measures. Particular attention was paid to improvements in shoulder range of motion (abduction and external rotation), radiological correction of glenoid version, and reported Mallet scores. Complications and secondary procedures were also recorded when available.

## 3. Results

Following the rigorous selection process, a total of three studies met the inclusion criteria and were included in this qualitative analysis. Collectively, these studies reported on 94 pediatric patients diagnosed with brachial plexus birth injury (BPBI) and treated surgically with glenoid osteotomy, either as an isolated procedure or in combination with additional surgical interventions.

### 3.1. Patient Demographics and Surgical Context

The included patients ranged in age from 3 to 18 years at the time of surgery. The gender distribution was slightly skewed toward males, although not all studies reported gender breakdown explicitly. The involved limb was the right side in most cases, consistent with the higher prevalence of right-sided BPBI due to delivery mechanics.

Prior treatments included early conservative management (physiotherapy, splinting) and, in some cases, previous surgical procedures such as tendon transfers or subscapularis release. These details are summarized in Table 1, which includes demographic characteristics, previous treatments, and the side affected.

### 3.2. Surgical Techniques

Out of the 94 patients, 65 underwent glenoid osteotomy in conjunction with tendon transfers (Table 2)—most commonly latissimus dorsi and teres major transfers—performed either simultaneously or in staged procedures. In terms of surgical technique, the glenoid osteotomy involved reorienting the glenoid cavity to decrease retroversion, with the use of structural bone grafts from donor sites including the scapular spine, coracoid process, and iliac crest. Fixation methods were not consistently reported, and there was some variability in osteotomy location and graft configuration.

### 3.3. Functional Outcomes

**Figure 2:**
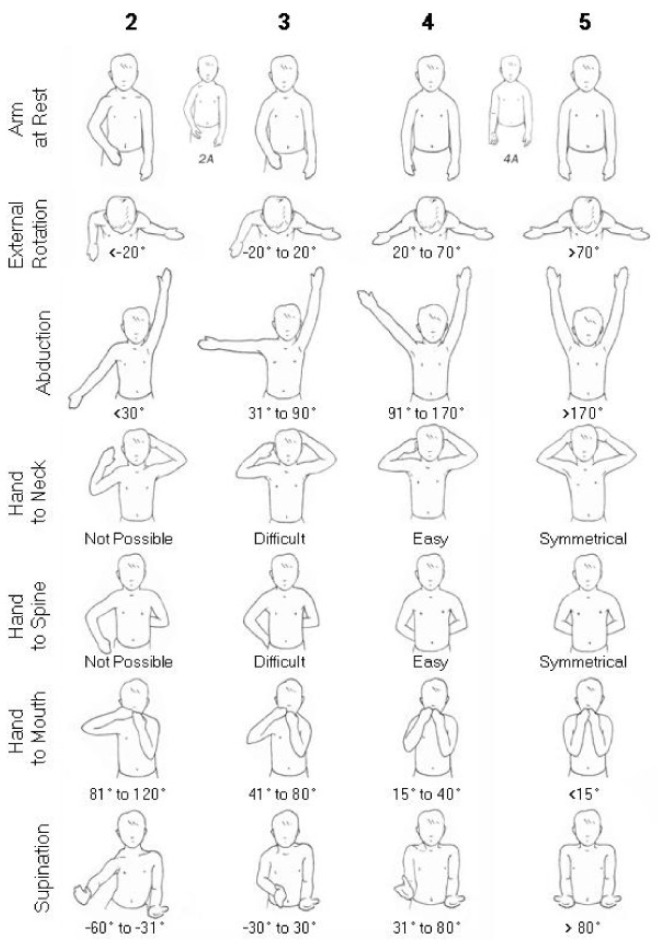
Mallet score.

All included studies evaluated functional outcomes using the Mallet score, a well-established clinical tool that assesses active shoulder function through various motions such as abduction, external rotation, hand-to-mouth, hand-to-neck, and hand-to-back movements. Improvements were noted in the total Mallet score (Figure 2) as well as in the abduction and external rotation subscores. These data are detailed in Table 3, highlighting pre- and postoperative values when available.

Although all children underwent advanced imaging (MRI or CT) pre- and postoperatively, only one study quantified the angle of glenoid retroversion. In that cohort, the mean retroversion decreased from 32° preoperatively to 6° postoperatively, yielding an average correction of 26°, indicating significant structural realignment.

### 3.4. Limitations in Data Reporting

Importantly, none of the included studies provided long-term follow-up beyond two years, and only one article reported on postoperative complications, which were limited and included graft displacement and transient pain. No cases of infection, permanent loss of range of motion, or neurovascular injury were documented in the available data.

Because of the small sample sizes, heterogeneous surgical techniques, and limited follow-up periods, a formal statistical analysis or pooled comparison of outcomes was not feasible.

## 4. Discussion

Brachial plexus birth injury (BPBI) represents a significant perinatal pathology, which, although relatively uncommon in terms of incidence, can lead to profound and enduring functional limitations of the upper extremity. Among the most debilitating sequelae associated with BPBI is the muscular imbalance that develops between the internal and external rotators of the shoulder. This disparity frequently progresses into a cascade of biomechanical disruptions that culminate in secondary structural abnormalities, most notably shoulder dysplasia (SD). Such deformities, it is important to note, are not merely anatomical in nature; they also carry substantial functional implications. These may severely restrict joint mobility and substantially diminish the quality of life during critical periods of growth and neuro-musculoskeletal development. As the articular surfaces remain incongruent during growth, the glenoid undergoes pathological remodeling characterized by flattening, retroversion, or biconcave facet formation. These deformities, once established, can substantially limit external rotation, shoulder abduction, and overall arm function [9,10,11].

Over the years, a range of both conservative and operative strategies has been proposed for managing SD. Despite these efforts, the optimal therapeutic pathway remains the subject of ongoing clinical discussion and debate. Traditional approaches have largely centered on soft tissue releases and humeral derotational osteotomies, primarily aiming to restore muscular balance and compensate for altered joint orientation. However, these interventions often prove inadequate in directly addressing the fundamental skeletal deformities of the shoulder, particularly those involving the glenoid fossa.

This is especially true in intermediate stages of dysplasia, such as Waters grades III and IV, in which the articular surfaces, while abnormal, retain a degree of plasticity that may respond favorably to corrective interventions associating the classical approach on soft tissues with skeletal procedures [12,13,14].

In this context, glenoid osteotomy has gained attention as a potentially more direct and structurally focused surgical approach. By physically reorienting the glenoid cavity of the scapula, this technique aims to correct the pathologic retroversion commonly seen in BPBI-induced SD. This reorientation may, in turn, mitigate posterior subluxation and help restore congruity to the glenohumeral joint. Conceptually, this method draws parallels with established orthopedic interventions in developmental dysplasia of the hip (DDH), where acetabular osteotomy is employed to improve femoral head containment and encourage more normal joint morphology. Applying a similar biomechanical logic to the shoulder, it is hypothesized that repositioning the glenoid could enhance humeral head coverage and foster a more physiological environment conducive to remodeling, particularly in skeletally immature patients.

Nevertheless, several critical anatomical and biomechanical differences between the shoulder and hip must be considered. The shoulder, as a non-weight-bearing joint, relies extensively on dynamic muscular stabilization rather than bony congruence. As a result, the predictability of outcomes following purely structural interventions such as osteotomy may be inherently lower compared to the hip. Furthermore, while the pediatric shoulder does possess some capacity for remodeling, this potential diminishes significantly with age, making the timing of surgical intervention a key determinant of its long-term success.

While the limited data suggests a general trend toward functional improvement—particularly in shoulder abduction and external rotation, as measured using the Mallet classification—there are important caveats.

Most surgical interventions were combined with adjunct procedures such as tendon transfers or open joint reductions. Consequently, isolating the specific contribution of the osteotomy to functional outcomes becomes analytically challenging [15,16,17,18].

Another major limitation in the existing body of literature is the short duration of follow-up. The majority of available data pertains to clinical outcomes measured within two years of surgery—an interval too brief to fully assess the durability of improvements or the long-term influence of glenoid realignment on the progression of dysplasia. Equally concerning is the absence of quantitative assessments regarding key parameters such as joint congruency, physeal adaptation, or progressive remodeling of the glenoid cartilage, particularly as visualized through longitudinal MRI or CT imaging [19,20,21,22].

The ideal candidate for glenoid osteotomy remains insufficiently defined. While it is theoretically posited that younger patients with moderate dysplasia (Waters III–IV) might benefit the most, due to their higher remodeling potential, the existing studies have included patients up to 18 years of age [23,24,25,26,27,28].

It is plausible that older children and adolescents might experience less substantial anatomical remodeling and derive benefits primarily from improved mechanical alignment rather than biological correction. Future investigations should seek to stratify outcomes based on age, skeletal maturity, and dysplasia severity, all of which are likely to be influential variables in both surgical feasibility and efficacy.

From a technical perspective, considerable variability in surgical approach exists. Differences were noted in the osteotomy technique employed, the site of autologous bone graft harvest (e.g., scapular spine, coracoid process, iliac crest), and the extent to which the osteotomy was performed as an isolated procedure or in conjunction with others. This heterogeneity complicates the interpretation of clinical outcomes and further emphasizes the pressing need for standardized operative protocols, ideally supported by multicenter collaboration [29,30,31].

Regarding safety, the procedure appears to have an acceptable risk profile based on current data. Reported complications were relatively infrequent and generally minor, including transient postoperative pain and isolated graft-related concerns.

Another relevant consideration is the potential synergistic benefit of combining glenoid osteotomy with soft tissue procedures. Restoring more physiological joint alignment may enhance the mechanical advantage of transferred tendons, allowing them to function more effectively within their new arc of motion. While this hypothesis remains largely speculative, it offers an intriguing avenue for future randomized studies comparing combined versus isolated interventions in similarly staged patients [32,33,34,35].

To truly advance this promising yet under-investigated field, future research should prioritize the following:

- The design and execution of prospective, multicenter trials involving large, diverse pediatric cohorts;
- The development of uniform imaging and clinical assessment protocols, including the use of 3D imaging for surgical planning and outcome evaluation;
- The integration of multidimensional outcome measures, such as the Mallet scale, objective strength assessments, and patient- or parent-reported quality-of-life instruments;
- The implementation of longitudinal follow-up extending into late adolescence or even early adulthood.

Modern imaging techniques—including high-resolution MRI and CT-based 3D reconstructions—should play a central role not only in preoperative planning but also in assessing postoperative outcomes, including changes in glenoid version, humeral head containment, and articular cartilage health. Likewise, functional evaluation should extend beyond passive range of motion, incorporating objective strength testing and real-world assessments of daily activity and independence.

In conclusion, glenoid osteotomy stands as a theoretically sound and surgically feasible technique with the potential to meaningfully improve outcomes in selected patients suffering from shoulder dysplasia secondary to BPBI. Despite encouraging early results—particularly regarding improvements in shoulder abduction and external rotation—the lack of high-quality, long-term evidence limits its current role to that of a specialized, adjunctive procedure performed in expert centers. Given its conceptual alignment with successful interventions in other pediatric dysplasias, further rigorous investigation is not only justified but necessary.

## Figures and Tables

**Table 1:** Patient demographics and number of previous surgeries.

	N° of Patient	Median Age	Gender M/F	Side R/L	Previous Surgery
Zargarbashi 2023	33	27.5 +/− 14 months (range 0.8–4.4)	18 vs. 15	12 vs. 21	Not included in the study
Dodwell 2012	32	6.8 years (range 2.1–16.2)	13 vs. 19	32 vs. 0	21
Di Mascio 2011	29	5 years (range 1–18)	12 vs. 17	14 vs. 15	14

**Table 2:** Different types of tendon transfers.

	Anterior Release	LD Transfer	LD and Teres Major Transfer	LD and Teres Minor Transfer	Lower Trapezium Transfer
Zargarbashi 2023	33	3	0	17	13
Dodwell 2012	32	0	32	0	0
Di Mascio 2011	28	0	0	0	0

**Table 3:** Mallet score and subscores.

	Pre-Mallet Score	Post-Mallet Score	Pre-Abduction	Post-Abduction	Pre-External Rotation	Post-External Rotation
Zargarbashi 2023	13.5 +/− 1.02 degrees	18.91 +/− 1.50 degrees	86.61 +/− 17.17 degrees	155 +/− 20.99 degrees	9.26 +/− 7.50 degrees	43.23 +/− 7.16 degrees
Dodwell 2012	11.05 degrees	15.04 degrees	104 degrees	129 degrees	66 degrees	98 degrees
Di Mascio 2011	8.25 degrees	11.75 degrees	132 degrees	156 degrees	1 degrees	56 degrees

## Data Availability

All the analyzed data all reported in this paper.

## Abbreviations

The following abbreviations are used in this manuscript:

SD	Shoulder Dysplasia
BPBI	Brachial Plexus Birth Injury
CT	Computed Tomography
MRI	Magnetic Resonance Imaging
DDH	Developmental Dysplasia of the Hip
LD	Latissimus Dorsi
CA	Chiara Arrigoni
RF	Roberto Facchi
NC	Nunzio Catena
